# Delay in Publication

**Published:** 1868-09-15

**Authors:** 


### *EDITORIAL.*

☞ Circumstances, over which we had no control, have delayed the present number a few days. We shall be up to time again at our next issue. Owing to the pressure upon our space, foreign items are necessarily postponed until next number.

Attention is especially directed to the exhaustive essay on the pathogenesis of disseminated chronic pneumonia and of pulmonary tubercles, by PROF. LEBERT, now passing through our pages. The name of its illustrious author is sufficient to vouch for its entire reliability. The conclusion will be given within the subsequent or next ensuing issue. It alone, we feel warranted in saying, is worth more to the thinking practitioner, than a year's subscription price of the JOURNAL.

